# Patient-Friendly Real-Time Optical Tomographic Imaging System (LOTIS) for Lupus Arthritis

**DOI:** 10.3390/bios16040184

**Published:** 2026-03-24

**Authors:** Moegammad A. Bardien, Lara Pinar, Alessandro Marone, Alberto Nordmann-Gomes, Leila Khalili, Stephen Suh, Stephen H. Kim, Anca D. Askanase, Andreas H. Hielscher

**Affiliations:** 1 Department of Biomedical Engineering, Tandon School of Engineering, New York University, Brooklyn, NY 11201, USA; am11201@nyu.edu (A.M.); ahh4614@nyu.edu (A.H.H.); 2 Department of Medicine—Rheumatology, Columbia University, Irving Medical Center, New York, NY 10032, USA; an3363@cumc.columbia.edu (A.N.-G.); lk2482@cumc.columbia.edu (L.K.); sks2231@cumc.columbia.edu (S.S.); ada20@cumc.columbia.edu (A.D.A.); 3 Department of Medicine—Department of Radiology, Grossman School of Medicine, New York University Langone Health, New York, NY 10016, USA

**Keywords:** systemic lupus erythematosus, optical tomography, joint inflammation, wearable device, hemodynamic monitoring

## Abstract

Systemic lupus erythematosus (SLE) frequently presents joint pain and stiffness, yet clinicians lack an objective, rapid method to quantify joint inflammation at the point of care. We introduce the Lupus Optical Tomography Imaging System (LOTIS), a wearable near-infrared (NIR) device that performs real-time three-dimensional tomographic imaging of hemodynamic changes in finger joints. LOTIS was developed to address key limitations of our earlier Flexible Optical Imaging System (FOIS), including mechanical fragility, high noise levels, single-joint acquisition, and slow reconstruction times. The new system integrates modular, mechanically robust optical patches with on-sensor digitization and a computationally efficient, non-iterative multispectral reconstruction algorithm to produce frame-by-frame maps of hemoglobin concentration. In a preliminary study using a standardized venous-occlusion protocol, LOTIS differentiated SLE-affected joints from those of healthy controls. Diseased joints exhibited blunted and spatially diffuse hemodynamic responses, whereas healthy joints showed localized and robust changes. These results demonstrate that LOTIS provides an operator-independent, patient-friendly method for quantifying joint-specific hemodynamic changes in real time, offering strong potential as a clinical tool for objective assessment and longitudinal monitoring of lupus arthritis.

## 1. Introduction

Systemic Lupus Erythematosus (SLE) is a chronic autoimmune disease that affects various organs in the body. The Centers for Disease Control and Prevention (CDC) estimates 204,000 individuals live with SLE in the US [1]. While the disease can affect many organs and systems, including skin, kidneys, cardiovascular system, and central nervous system, one of the most common manifestations is joint involvement. In particular, joints of the hands, wrists, elbows, and knees [2] are most often affected. SLE causes joint manifestation in 91% of patients that can affect their ability to complete activities of daily living [3,4]. A significant confounding factor is the frequent disconnect between subjective patient-reported symptoms and objective clinical findings. Patients may report debilitating pain and stiffness, while physical examination reveals only mild or no signs of synovitis [5]. This discrepancy renders clinical assessment of arthritis difficult and hinders its standardization.

The current framework for managing SLE arthritis relies on a combination of clinical findings, imaging modalities, and patient reports, each with its own limitations, which create a gap in both patient care and clinical research.

For monitoring disease activity, clinicians and researchers use disease activity indices such as the Systemic Lupus Erythematosus Disease Activity Index (SLEDAI-2K), the British Isles Lupus Assessment Group index (BILAG-2004), Easy-BILAG, and tender and swollen joint counts [6,7,8]. The main drawback of SLEDAI is its inability to capture changes in symptoms within an organ system [9]. While BILAG is more comprehensive than SLEDAI, it requires training and more time to complete and involves laboratory tests, making it less practical for routine clinical use [10]. All these indices, in their arthritis items, rely on subjective physician assessments that introduce significant inter-rater variability. Collectively, these limitations create a significant gap in clinical management, leaving clinicians and researchers without a reliable method for tracking disease progression and response to therapy.

Imaging techniques have been used to overcome this subjectivity; however, current techniques have significant drawbacks. Conventional radiography techniques, such as X-ray, are ill-suited to detecting soft tissue inflammation present in SLE and can only detect late-stage bone damage, which may not be present or appear too late for effective intervention [11]. Musculoskeletal ultrasound (MSK-US) and magnetic resonance imaging (MRI) offer better soft-tissue visualization but have their own limitations. Due to the chronic nature of SLE and the cost and availability of MRI, disease management and tracking with MRI are seen as impractical and costly [12]. Additionally, while MRI is well-established for rheumatoid arthritis (RA) because RA has clear, structural hallmarks that MRI can capture, the same is not the case for lupus arthritis [11]. Classic lupus arthritis can present with pain, stiffness, and swelling, but without the same degree of synovial hypertrophy or erosions that MRI detects so clearly in RA. Therefore, studies that use RA MRI criteria to score lupus arthritis showed mixed results [13].

MSK-US is highly operator-dependent, and the interpretation of the data can be subjective [12]. Furthermore, like MRI, the use of MSK-US is not as well established in SLE as it is in rheumatoid arthritis (RA). There are no universally accepted, lupus-specific scoring criteria, and many practices are inherited from their use in RA [14].

Diffuse Optical Tomography (DOT) is an emerging, non-invasive imaging modality that holds significant promise for addressing the existing gap in arthritis assessment [15]. DOT utilizes near-infrared (NIR) light, in the wavelength range of 650 nm to 900 nm, which can penetrate several centimeters into biological tissue, allowing for the interrogation of joint structures [16]. The fundamental principle of DOT involves illuminating the tissue and measuring the intensity of transmitted and reflected light. Light propagation in biological tissue depends on its underlying optical properties, which are determined by composition and structure [17]. Differences in chromophore concentrations result in distinct absorption characteristics, enabling noninvasive derivation of physiological parameters. By measuring transmitted and reflected light intensities from multiple source–detector (SD) pairs at the tissue surface and applying image reconstruction algorithms, it is possible to reconstruct three-dimensional maps of tissue chromophore concentrations—such as total hemoglobin (THb). These maps reveal structural and functional information in tissues such as joints [18].

Building on the established potential of optical imaging for RA [19], our group previously developed the Flexible Optical Imaging System (FOIS), a first-generation, wearable device specifically designed for the assessment of arthritis in the finger joints of SLE patients [20]. Despite this initial promise, the FOIS design had several key limitations that hindered its clinical translation. First, the system acquired data from a single finger at a time, significantly increasing the time required to complete measurements. Second, the placement of the probe around the finger was performed by manually wrapping a strip of flexible circuitry around the joint. This procedure could take up to a minute and needed to be repeated several times if no good contact was made between the probe and the finger. Third, the system’s hardware architecture resulted in measurements that were often noisy, yielding signal-to-noise ratios (SNR) of 7 dB for the noisiest source detector pairs. Finally, the image reconstruction code used to analyze the data took 20 min per finger. Therefore, it was impossible to inspect the image quality in real-time, and in some instances, patients had to be called to repeat the measurements.

To overcome these specific limitations, we have developed the Lupus Optical Tomography Imaging System (LOTIS), a next-generation, high-speed, high-density optical imaging device. LOTIS enables the simultaneous collection of data from multiple fingers, significantly reducing image acquisition time. A spring-loaded clamp design simplifies the probe placement on the finger, and a new hardware architecture produces higher-quality data. Additionally, a new image reconstruction code enables the production of images in real-time. This paper presents the design, fabrication, and initial testing of the device in human subjects.

## 2. Materials and Methods

### 2.1. Instrumentation

**Figure 1:**
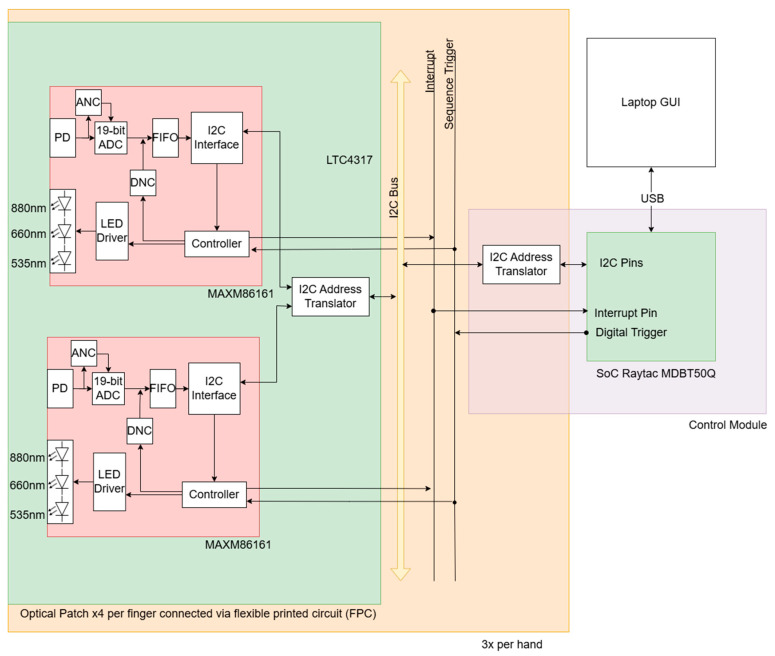
A system block diagram of the Lupus optical imaging system showing interfaces between key components, including MAXM86161 sensors and the MDBT50Q system on chip (SoC). Each sensor integrates a photodiode (PD), a 19-bit analog-to-digital converter (ADC) with ambient and digital noise cancellation (ANC and DNC), and an LED driver. Data is managed via an internal first-in-first-out (FIFO) buffer and I2C interface. Multiple optical patch units connect via a flexible printed circuit (FPC) and LTC4317 I2C address translators to a central I2C bus that carries interrupt and sequence-trigger signals. The control board coordinates these signals with the SoC, transmitting data to a laptop graphical user interface (GUI) via USB.

The hardware architecture of LOTIS follows a modular, hierarchical design, scaling from individual optical components up to the multi-finger system level (Figure 1). The core sensing units are the MAXM86161 optical source-detector modules (Analog Devices, Wilmington, MA, USA). Each module is an integrated package containing three light-emitting diodes (LEDs) (880 nm, 660 nm, and 535 nm; only 660 nm and 880 nm are used in this study) and one photodiode (PD). Within this single module, the LED-to-PD distance is fixed at 2 mm. The modules feature built-in analog-to-digital converters (ADCs), ambient and digital noise cancellation (ANC and DNC), first-in-first-out (FIFO) memory registers, and integrated I2C communication.

**Figure 2:**
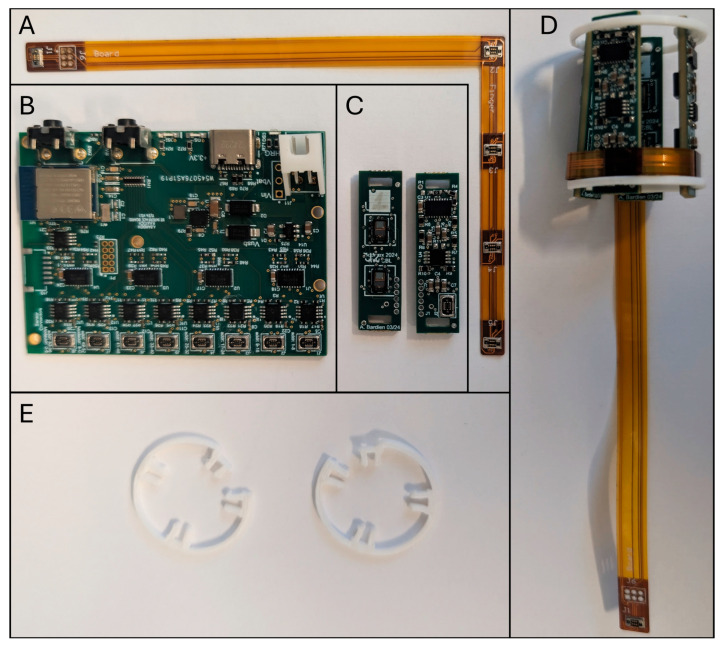
The components that make up the LOTIS, including the flexible printed circuit (FPC) connecting band (**A**), the control module (**B**), the front and back of two optical imaging patches (**C**), a flexible fixation band attached to a flexible band and four optical imaging patches (**D**), and the 3d printed fixation clips (**E**).

Two optical modules are mounted onto a single rigid printed circuit board to form an optical imaging patch (Figure 2C), with the two modules fixed at a distance of 10 mm apart. Each patch also includes a local I2C address translator (LTC4317, Analog Devices). To monitor a single finger, four of these patches are mounted onto a flexible printed circuit (FPC) connecting band (Figure 2A) at 15 mm intervals. While the individual patches are rigid to maintain consistent intra-patch geometries, the FPC band connecting them is highly flexible (Figure 2D). This allows the entire assembly to wrap conformally around the proximal interphalangeal joint. The assembly is secured to the finger using 3D-printed adjustable fixation clips (Figure 2E). Because finger circumferences vary between patients, these clips allow the flexible band to be tightened and locked into place. This adjustment mechanism ensures that despite the rigid nature of the individual patches, the overall flexible structure adapts to the finger so that all LEDs and PDs maintain direct, continuous contact with the skin throughout the measurement.

A central control module (Figure 2B) connects to up to eight FPC bands, allowing simultaneous imaging of multiple fingers. For this study, three fingers were monitored using a total of 12 patches. The control module houses a system-on-a-chip (SoC; Raytac MDBT50Q, Raytac Corporation, New Taipei City, Taiwan), power regulation, and a secondary addressing layer to uniquely identify all patches across multi-finger configurations (see Supplementary Materials Text S1 and Table S1 for a detailed description). A single USB cable connects the module to the computer, handling both data transfer and power. The control module also includes built-in Bluetooth and battery charging circuitry for future wireless data acquisition.

### 2.2. Data Acquisition and Firmware

Upon initialization, the system begins with a scan to detect the attached optical patches and their associated I2C addresses. Each patch is assigned to a channel associated with a specific finger. An interrupt handler, triggered by the interrupt pins of the MAXM86161s, is initialized. Once the initialization process is complete, the system sends a ready signal to the connected graphical user interface (GUI) and then waits for a start acquisition signal in return.

When the start signal is received, data acquisition begins. During acquisition, each MAXM86161 can be in one of two states: active wake or passive wake. On each finger, only one module is in the active wake state. Upon receiving a trigger signal, a module in active wake will have its LEDs sequentially activated. Synchronously, modules in the passive wake state will measure the light emitted by the active modules. Once the active module has completed the sequence of lighting its LEDs and the passive modules have recorded measurements, an interrupt signal is sent to the control board. The active module is then set as passive, and the next module is set to the active wake state. The trigger signal is then sent again, and the process repeats. When the last module on a finger has completed its active wake sequence, the first module is then set as active, and the process repeats. Once a stop signal is sent from the GUI, the sequence is reset, and the system waits for a new start signal to begin a new set of data acquisition. This usually occurs between measurements of alternate hands. At an acquisition rate of a minimum of 1.25 Hz (lower capacity configurations have higher acquisition rates), 16 LEDs and 8 detectors acquire 128 measurements per finger per frame (a full set of measurements from all source detector pairs at a particular time point; frame time = 0.8 s per frame). If three proximal interphalangeal joints on a hand are imaged simultaneously, 384 measurements are acquired per frame.

### 2.3. Real-Time Reconstruction Algorithm

#### 2.3.1. Background

Traditional dynamic DOT can be performed using either linear or nonlinear models. Linear models are simple to implement and computationally fast but are only valid for small absorption changes [21]. In contrast, nonlinear methods offer greater accuracy over a wider range of changes but are computationally expensive due to their iterative optimization procedures, which require repeated solving of the light propagation model [22].

To address these limitations, our group recently developed the Sensitivity-Equation-Based Non-Iterative Sparse Optical Reconstruction (SENSOR) algorithm. SENSOR reformulates the nonlinear inverse DOT problem into a linear one, eliminating the need for iterative forward computations and dramatically reducing computational complexity. SENSOR has demonstrated high spatio-temporal resolution and accuracy compared to traditional linear perturbation models [23]. However, the original SENSOR was applied only to the reconstruction of absorption coefficients in static imaging settings and has not been explored for multispectral dynamic DOT imaging of tissue chromophores.

Here, we present a real-time multispectral image reconstruction method that builds upon the SENSOR framework to derive tissue optical properties, including chromophores such as oxygenated (HbO_2_) and deoxygenated (Hb) hemoglobin, as well as total hemoglobin concentration (THb). Our work extends SENSOR in two key aspects: (1) incorporation of a multispectral model that uses data from multiple wavelengths to directly reconstruct concentrations of multiple chromophores, whereas the original SENSOR was limited to imaging absorption coefficients alone; and (2) implementation of a discrete cosine transform (DCT)-based image compression that regularizes the inverse problem more effectively than traditional Tikhonov-type regularizations. This improves the reconstruction speed and accuracy by reducing the degrees of freedom compared to the element-by-element approach in the original SENSOR. The reconstruction algorithm is implemented in MATLAB R2025a, with matrix operations and numerical optimization performed using built-in functions and the MATLAB App Designer.

#### 2.3.2. Methods

Forward model of light propagation in tissue

In continuous wave (CW) dynamic DOT, the forward model of light propagation in scattering dominant biological tissue can be well described with the diffusion equation:

(1) $$\nabla D\left(r\right)\nabla \phi \left(r\right)-\mu_{a}\phi \left(r\right)+q\left(r\right)=0$$

where *ϕ*(*r*) is the fluence in units of Wcm^–2^, r represents the position vector which denotes the specific 3D location $(x,\;y,\;z),\;\;D={\left(3\left(\mu_{a}+\mu_{s}^{\prime }\right)\right)}^{-1}$ is the diffusion coefficient, $\mu_{a}$ is the absorption coefficient and *q* denotes an internal source of light [24].

The total absorption coefficient is linearly related to chromophore concentrations through the Beer–Lambert law, as:

(2) $$\mu_{a}\left(\lambda \right)=\sum_{i=1}^{N_{c}}\varepsilon_{i}\left(\lambda \right)C_{i}$$

where $\varepsilon_{i}\left(\lambda \right)$ and $C_{i}$ represent the extinction coefficient and concentration of the i-th chromophore, respectively. $N_{c}$ denotes the number of chromophores contributing to absorption at wavelength λ [25,26,27,28].

Given the discretized forward model of Equation (1) given by A(μ)ϕ=b, a generalized sensitivity formulation is derived to estimate the exact variation in measurements resulting from arbitrary changes in system properties, following a generalized perturbation and adjoint theory. This formulation accounts for both the direct (first-order) effect due to changes in the medium’s optical properties Δμ and the indirect (second-order) effect caused by variations in light intensity Δϕ.

By introducing an adjoint variable, SENSOR expresses the change in measured detector signals as a function of both the reference fluence and the perturbation in optical properties. This approach generates a non-truncated sensing matrix which incorporates all relevant contributions. With the reference measurement $\bar{z}$, the target measurement z, and the precomputed sensing matrix $S_{nt}$, the change in optical properties Δμ can be obtained by solving a least-squares problem:

(3) $$\Delta \mu =\underset{\Delta \mu }{\mathrm{argmin}}{\left|\left|\Delta Z-S_{nt}\Delta \mu \right|\right|}_{2}^{2}$$

where $\Delta Z=z-\bar{z}\in R^{N_{s}M\times 1}$ is the measurement change at $N_{s}$ source illuminations at locations $r_{i}\left(i=1,\ldots ,\;N_{s}\right),$ M measurements at locations $r_{j}\left(j=1,\ldots ,\;M\right)$, $S_{nt}\in R^{N_{s}M\times N}$ is the nontruncated sensing matrix at N nodes, and $\Delta \mu \in R^{N\times 1}$ is the change in optical properties. For a full derivation of the variational relations and sensitivity formulation, see [23]. The SENSOR algorithm computes Δμ through the nontruncated sensing matrix without repeated solving of the forward model, which leads to great improvements in computational efficiency while maintaining accuracy [29].

Multispectral SENSOR inverse model and DCT image compression

We extend the SENSOR formulation to incorporate multiple wavelengths. In the multispectral model, we directly reconstruct chromophore concentrations from measurements at $N_{\lambda }$ wavelengths simultaneously within the inverse model. To this end, we reformulate the original SENSOR model to allow for direct imaging of chromophore changes as:

(4) $$C_{i}=\underset{\Delta C}{\mathrm{argmin}}{\left|\left|\Delta Z^{\lambda }-S_{nt}^{\lambda }\Delta C\right|\right|}_{2}^{2}$$

where $\Delta Z^{\lambda }=z-\bar{z}\in R^{N_{s}N_{\lambda }M\times 1}$ is the measurement change evaluated at $N_{\lambda }$ wavelengths, $S_{nt}^{\lambda }\in R^{N_{s}N_{\lambda }M\times N}$ is the nontruncated sensing matrix computed with respect to the concentrations $C_{i}$ of chromophores, and $\Delta C\in R^{N\times 1}$ is the change in chromophore concentrations. Note that $\Delta Z^{\lambda }$ and $S_{nt}^{\lambda }$ incorporate all wavelengths that were used.

DOT presents a strongly ill-posed inverse problem due to the limitations of the measured data. In practice, the number of available measurements ($m=N_{s}N_{d}$) depending on the number of sources ($N_{s}$) and detectors ($N_{d}$) is typically much smaller than the number of unknown parameters (n), i.e., m≪2n. This imbalance leads to reconstructions that are highly unstable with respect to measurement noise, making regularization essential for obtaining reliable solutions. Regularization is generally realized by modifying the objective function with additional stabilizing terms [24]. Classical implementations often rely on Tikhonov-type operators to impose smoothness constraints [30].

In this work, we employ a DCT–based strategy. This is widely used in JPEG compression and exploits the sparsity of spatial-frequency representations to reduce the dimensionality of the problem. By expressing the original high-dimensional image with only a limited set of coefficients, the DCT method preserves essential structures while lowering the number of unknowns [31]. Hence, DCT-based regularization offers a robust and efficient framework for stabilizing DOT reconstructions, different from traditional techniques.

With DCT, the spatial distributions of unknown changes in chromophore concentrations ${∆C}_{i}(x,y,z)$, given as a function of spatial coordinates (x,y,z), are transformed into a linear combination of discrete cosine basis functions and their corresponding coefficients in the spatial-frequency domain, given in a compact matrix-vector form as:

(5) $$∆C_{i}=Bw_{\mu }$$

where $w_{\mu }$ is the vector of inverse variables, $∆C_{i}$ is the vector containing the DCT coefficients $C_{mnk}$ and B is the DCT basis function matrix with entries $B_{i}{,}_{\left(mNK+nK+k+1\right)}=cos\left(\frac{m\pi x}{L_{x}}\right)cos\left(\frac{n\pi y}{L_{y}}\right)cos\left(\frac{k\pi z}{L_{z}}\right)$. By incorporating the DCT basis expansion into the original forward model, the inverse problem is reformulated to solve for the coefficients $w_{\mu }$ instead of $∆C_{i}$. Consequently, the sensing matrix of the measurements with respect to the DCT coefficients, denoted ${S_{nt}}_{DCT}$, is derived using the chain rule:

(6) $${S_{nt}}_{DCT}=\frac{\partial z}{\partial w_{\mu }}=\frac{\partial z}{\partial \mu }\frac{\partial \mu }{\partial w_{\mu }}=\frac{\partial z}{\partial \mu }\frac{\partial }{\partial w_{\mu }}\left(Bw_{\mu }\right)={S_{nt}}_{\mu }B$$

where z is the measurement vector and ${S_{nt}}_{\mu }$ is the original sensing matrix. With the new DCT-based sensing matrix, the inverse problem is now to solve Equation (4) for the optimal DCT coefficients $w_{\mu }$:

(7) $$\underset{w_{\mu }}{\min}{\left|\left|{S_{nt}}_{DCT}w_{\mu }-z\right|\right|}_{2}^{2}$$

The final step is mapping the coefficients back through the basis matrix to reconstruct the distribution of optical properties μ on the original unstructured grid by solving Equation (6). In effect, the DCT’s ability to compress the image into a few coefficients acts as its own form of regularization, making the problem more stable and eliminating the need for any additional, explicit regularization terms.

Calibration, preprocessing, standardized meshes

The raw measurements were smoothed with a Gaussian filter to suppress noise. To accelerate the reconstruction process, the framework was designed to automatically assign an appropriate mesh from a predefined set of standardized meshes based on the subject-specific input measurements (e.g., finger geometries).

The smoothed measurements for each source–detector pair (s,d) were calibrated following the standard procedure described in [32]:

(8) $$z_{s,d}^{calib}=\frac{z_{s,d}^{target}}{z_{s,d}^{baseline}}\times z_{s,d}^{reference}$$

where $z_{s,d}^{target}$ and $z_{s,d}^{baseline}$ denote the perturbed and baseline measurements for the reference medium with unknown optical properties, $z_{s,d}^{reference}$ represents the reference state of the medium with known optical properties, and $z_{s,d}^{calib}$ is the calibrated measurement which is used in the reconstruction algorithm. The calibration scheme eliminates system-related artifacts by referencing the target measurements against both experimental and simulated baseline data. This combination of calibration, preprocessing, and standardized meshing ensured both consistency across datasets and computational efficiency in subsequent image reconstructions.

### 2.4. GUI

**Figure 3:**
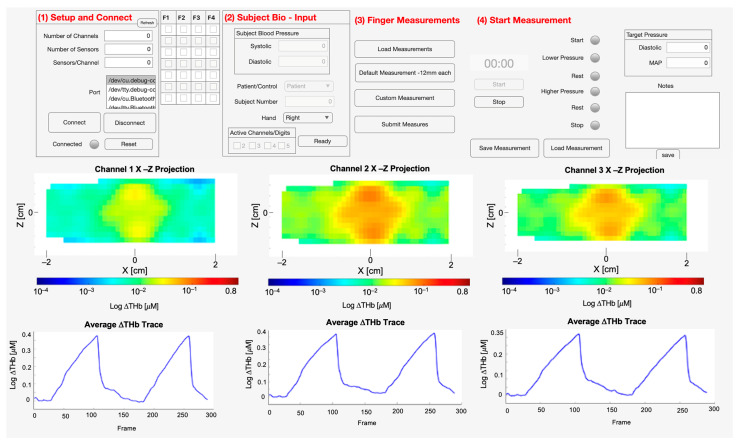
The LOTIS GUI.

The GUI (see Figure 3) facilitates a structured experimental protocol by integrating hardware control, subject data management, and real-time data visualization. Communication with the external sensing hardware is established via a serial port, with the application automatically detecting and listing available ports. Upon connection, the GUI queries the device for its hardware configuration, including the number of channels and sensors, which are displayed to the user for verification. A status lamp provides an immediate visual confirmation of the connection state.

The GUI features a dedicated panel for subject information, allowing for the entry of an anonymized unique subject number. A key component of this setup is defining the computational mesh used for image reconstruction. The user has the flexibility to (1) load a pre-existing, patient-specific mesh, (2) input the subject’s finger measurements to automatically generate a corresponding mesh, or (3) use default finger meshes provided by the system. Following mesh selection, the user inputs the subject’s systolic and diastolic values for the integrated blood pressure monitoring. The GUI then calculates and displays the Mean Arterial Pressure (MAP) and diastolic pressure, which is used to guide the experimental protocol.

Data acquisition is initiated and terminated via dedicated Start and Stop buttons. To ensure precise execution of the occlusion protocol, the GUI features a timed guidance system that actively prompts the experimenter to manually modify the sphygmomanometer cuff pressure. A digital timer tracks the elapsed time, while a series of colored lamps visually indicates when the experimenter must inflate or deflate the cuff for each distinct phase. Specifically, the GUI directs the experimenter through a predefined sequence: inflating the cuff to the calculated target diastolic pressure during the “Lower Pressure” phase, completely releasing the cuff pressure during an initial “Rest” phase, inflating the cuff to the MAP during a “Higher Pressure” phase, and finally, releasing the pressure again for a second “Rest” phase. The specific durations of these phases are detailed in Section 2.5. The application employs a parsing function that is triggered with each data transmission from the LOTIS, allowing it to parse incoming sensor data in real-time. Each data point is labeled with its corresponding channel, source, detector, and wavelength (WL).

Simultaneously, the GUI provides real-time visualization of both incoming raw data and the resulting hemodynamic changes. Dedicated axes display the reconstructed changes in hemoglobin concentration as both 3D X-Z projections and as mean signal traces over time for all three channels (joints). The operator also has the flexibility to select and display the raw data from any specific channel, source, detector, or wavelength combination. Finally, a separate text area is provided for capturing and saving any relevant notes, which are stored alongside the blood pressure data in a separate file.

The aggregated, labeled raw data stream is written to a primary text file. A robust file management system automatically generates this filename based on the subject’s metadata to ensure existing data is not overwritten. A complete record of the experiment is saved, which includes the raw data file, the final reconstructed values, the specific mesh information used for each channel, and a file containing the operator’s notes and the initial blood pressure data.

### 2.5. Measurement Protocol

To systematically assess microvascular reactivity and vascular compliance in the proximal interphalangeal (PIP) joints, we utilized a sequential venous and partial arterial occlusion protocol to provoke measurable vascular responses. Building upon our previous foundational work [20], the current protocol introduces patient-specific pressure targets to provide a more precise physiological assessment. The measurement protocol begins with taking the subject’s blood pressure measurement using a digital blood pressure monitor (OMRON BP742N, OMRON Healthcare, Kyoto, Japan). This data is then entered into the GUI, which in turn calculates the patient’s mean arterial pressure.

**Figure 4:**
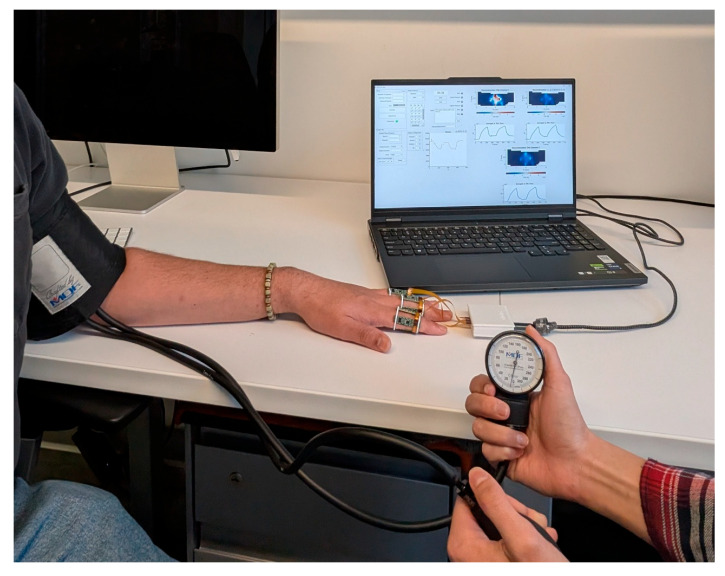
In situ view of the measurement setup, showing the subject in the correct measurement posture with LOTIS attached to the fingers and the sphygmomanometer on the upper arm.

The flexible bands with the attached optical patches are then fixed to one of the subject’s hands. In total, three fingers are measured per hand. The bands are attached so that the distal and proximal optical modules of each imaging patch are equidistant from the joint. The first patch is always positioned to be directly above the patient’s proximal interphalangeal joints. The patient is then instructed to sit with their arm fully relaxed upon a table with a 90-degree angle at the elbow. A sphygmomanometer is then attached to the patient’s upper arm. Figure 4 shows the measurement setup in situ.

**Figure 5:**
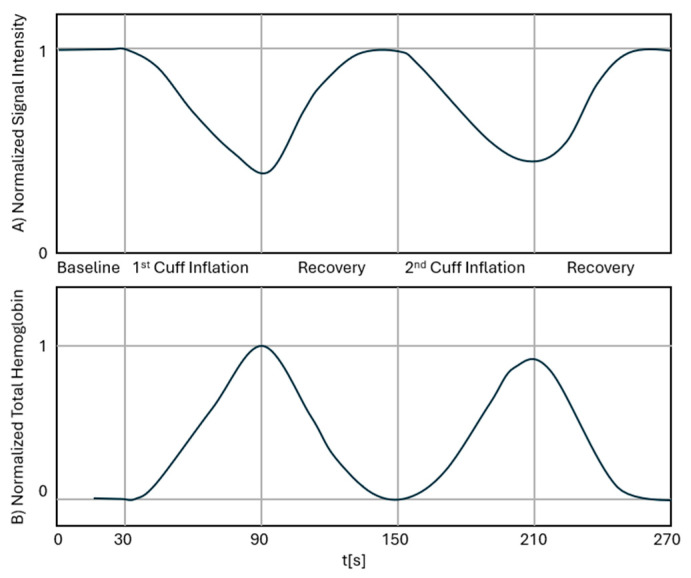
A typical signal trace measured by LOTIS for one source detector (**A**) and a typical reconstructed mean THb trace (**B**), showing significant events, including the baseline measurement, the sphygmomanometer cuff inflations, and the recovery periods.

Data acquisition is then initiated. A 30-s baseline measurement is recorded first. To induce a hemodynamic effect, the sphygmomanometer is inflated to the subject’s diastolic blood pressure, thereby inducing a venous occlusion. After 60 s, the pressure is released, and the measurement is continued for an additional 60 s, returning to baseline. The sphygmomanometer is then inflated to the patient’s mean arterial pressure, now inducing a venous occlusion and partial arterial occlusion. After 60 s, the pressure is released again, and for a further 60 s, the patient’s measurements are allowed to return to baseline. The measurement is then saved along with the patient’s blood pressure and any relevant notes taken during the measurement. The total measurement time is 4 min and 30 s. Figure 5 shows the expected shape of a typical raw signal trace for one source-detector pair (A) and a typical trace for the mean reconstructed THb in one finger (B). The process is then repeated for the contralateral hand.

### 2.6. Parameters of Interest

**Figure 6:**
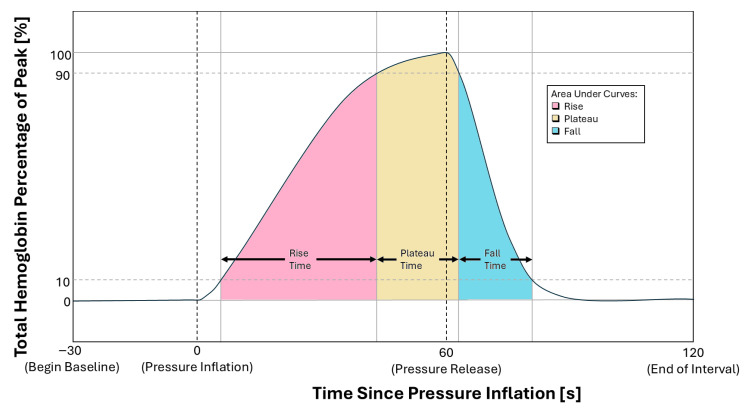
A representative reconstructed mean total hemoglobin (THb) trace during a single venous occlusion cycle, illustrating the extracted temporal parameters: Rise Time (RT), Plateau Time (PT), Fall Time (FT), and their respective areas under the curve (AUCR, AUCP, and AUCF).

In previous work, we demonstrated that temporal parameters from THb curves can effectively differentiate between diseased and healthy subjects in FOIS [20]. We analyzed the Rise Time (RT), the time taken from 10% to 90% of the signal peak; the Plateau Time (PT), the duration spent above 90% of the peak; and the Fall Time (FT), the time taken to descend from 90% to 10% of the peak. The accompanying Areas Under the Curve for each phase (AUCR, AUCP, AUCF) were also found to be strong differentiators. Figure 6 shows these parameters on a THb curve acquired from a patient for the one inflation-recovery cycle.

## 3. Results and Discussion

### 3.1. Comparison of LOTIS to FOIS

The LOTIS represents a significant design evolution from the proof-of-concept FOIS. The development of LOTIS was driven by the need to address specific limitations of the FOIS, primarily focusing on enhancing durability, signal integrity, and usability.

A primary limitation of the FOIS was its durability. The FOIS was constructed from a single, fully flexible PCB assembly, which, while highly compliant, subjected the solder joints of the optical modules to significant mechanical stress. This stress frequently led to component failure, necessitating constant repairs. Due to the integrated design, a single module failure could render an entire imaging band irreparable.

To overcome this, LOTIS employs a modular, hybrid-flex architecture. The optical imaging patches are fabricated on traditional rigid PCBs for maximum durability, while the interconnecting bands remain as flexible printed circuits (FPCs). This design isolates the rigid components from mechanical stress and allows for the independent replacement of patches and connecting bands, greatly improving serviceability.

To improve signal integrity, the LOTIS design minimizes the distance that analog signals must travel before digitization. The FOIS required raw, unconditioned analog signals from its OSRAM SFH7050 optical modules to travel across relatively long traces and a connector before being amplified and converted by a 16-bit ADC on a separate control board. This long analog signal path made the system susceptible to electromagnetic interference (EMI), as any noise introduced would also be amplified. As a result, the mean SNR across all SD-pairs increased from 17 dB to 23 dB, while the minimum and maximum SNR rose from 7 dB to 18 dB and 28 dB to 46 dB, respectively.

In contrast, LOTIS utilizes the Analog Devices MAXM86161 optical module. This component integrates the photodiode, a high-resolution 19-bit ADC, and communication circuitry into a single package. By performing the analog-to-digital conversion directly at the point of measurement, the system’s vulnerability to EMI is substantially reduced.

**Figure 7:**
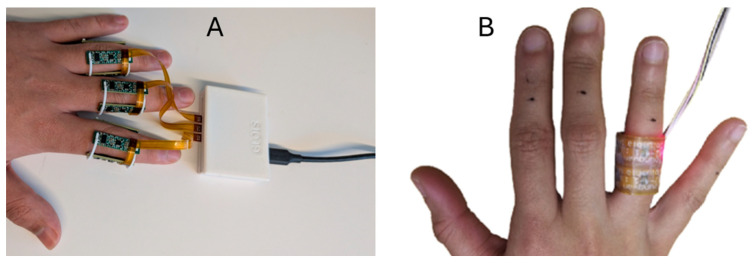
Image showing the LOTIS (**A**) and FOIS (**B**); adapted from [20] in situ.

Several key upgrades to the system’s ergonomics and usability were implemented in the LOTIS design. To improve usability, the system was expanded from the single-finger capability of the FOIS to allow for simultaneous multi-finger measurements. Furthermore, the Micropore surgical tape previously used for sensor attachment has been replaced with universal, 3D-printed flexible rings, which provide a more stable, comfortable, and repeatable patient interface. The number of wavelengths used was also refined based on findings from the FOIS. While the original system tested three wavelengths, the LOTIS protocol has been optimized to use only red (660 nm) and infrared (880 nm) light, as the high absorption of green light by biological tissue was found to yield data with a low signal-to-noise ratio, making it redundant for this application. Figure 7 shows a comparison of the LOTIS and FOIS in situ.

To complement the hardware upgrades, the LOTIS implements an advanced real-time reconstruction algorithm that extends the SENSOR framework. By incorporating a multispectral model for direct chromophore reconstruction and replacing standard regularization with a DCT-based strategy, we addressed the computation time and stability issues of previous methods. This software evolution significantly improves efficiency, enabling the system to achieve reconstruction times of just 0.5 s per frame without sacrificing accuracy.

**Figure 8:**
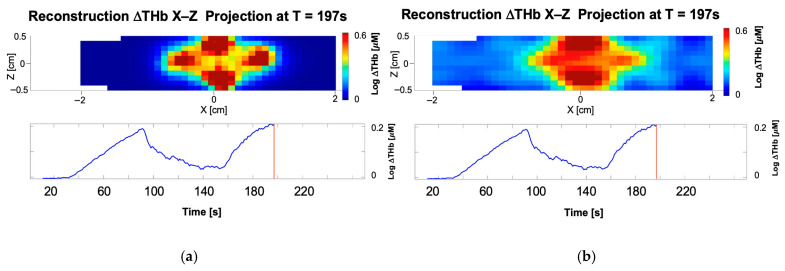
X-Z projection maps and averaged traces of hemoglobin concentration changes with (**a**) element-wise reconstruction and (**b**) DCT-based reconstruction.

Figure 8 compares the reconstruction results for hemoglobin concentration changes (ΔTHb) in a finger, obtained using conventional element-wise reconstruction and the proposed DCT-regularized approach with a 10 × 10 × 10 grid of spatial-frequency coefficients. The color-coded images represent X-Z projection maps, where the maximum value per pixel was selected across all Y-slices (Maximum Intensity Projection). In these maps, the *X*-axis represents the longitudinal length of the finger, while the *Z*-axis represents depth. The maps correspond to the rightmost time point (T = 197 s), indicated by the red vertical line in the temporal traces shown below them. The traces represent the mean change in hemoglobin averaged across all nodes in the volume at each specific timepoint relative to the baseline. Because the DCT-based strategy acts as a spatial regularization—redistributing values and smoothing extremes without altering the global total—the averaged temporal traces for both methods remain identical.

The element-based reconstructions are dominated by noise and artifacts, with higher intensity at the upper and lower edges of the projection which reflect the highest measurement sensitivities in the immediate proximity of the light sources and detectors (Figure 8a). In contrast, the DCT-based reconstruction yields a smoother and more accurate recovery of hemoglobin changes across the domain, delineating the expected physiological variations around the joints (Figure 8b). The regions with higher hemoglobin correspond to the veins surrounding the finger, while the central area shows smaller changes due to the presence of the bone.

This advanced performance stems from the DCT framework’s ability to represent essential features with a limited set of coefficients. By achieving this dimensionality reduction, the framework mitigates the inverse problem’s ill-posedness and provides effective built-in regularization. Consequently, the approach enhances accuracy and noise robustness while improving computational efficiency by significantly reducing the number of optimization parameters. This efficiency gain is reflected in the inverse model computation, as the DCT-based method requires only about half the computation time compared to the conventional element-wise formulation.

Key design differences between the two systems are summarized in Table 1.

### 3.2. Initial Human Measurements Demonstrating System Capability

LOTIS was evaluated in a small cohort of three healthy volunteers and three patients diagnosed with systemic lupus erythematosus (SLE) to demonstrate the system’s ability to acquire stable, interpretable hemodynamic measurements in human subjects. These measurements were intended solely to assess system feasibility, robustness, and physiological plausibility, and were not designed or powered to support clinical inference.

**Figure 9:**
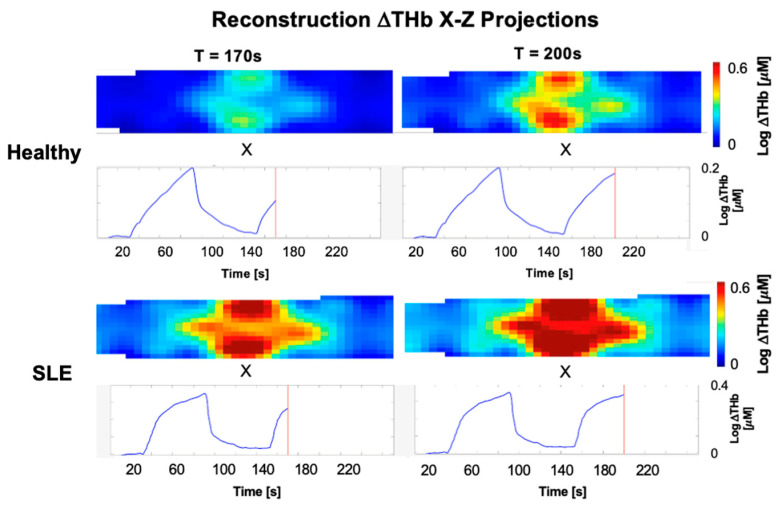
Representative X-Z projection maps and corresponding averaged temporal traces of hemoglobin concentration changes at selected time points (T = 170 s and 200 s) for healthy and diseased (SLE) joints.

Representative 3D X-Z projections and averaged time traces of THb concentration changes acquired during the venous occlusion protocol at selected time points are shown in Figure 9. The system was able to capture distinct hemodynamic profiles corresponding to different physiological states. In diseased joints, a larger area exhibits elevated hemoglobin concentration compared to the healthy joints which indicates that blood is pooling over a wider region during venous occlusion. In the context of SLE, this spatial expansion of hemoglobin can be indicative of underlying joint inflammation, which generally causes increased blood volume in the surrounding tissues. In contrast, healthy subjects show a more localized response with lower overall amplitudes for changes in hemoglobin. Beyond the spatial maps, the temporal traces in Figure 9 show clear differences between the healthy and diseased joints. First, the overall amplitude of the signal is higher in the SLE joints, peaking at approximately 0.35, compared to about 0.2 for the healthy joints. This higher peak directly matches the larger area of blood pooling seen in the spatial images. However, the shapes of the curves are also noticeably different. The healthy traces have a distinct triangular shape, rising steadily during the occlusion to a sharp peak before coming back down. In contrast, the SLE traces show a blunted, rounded waveform.

To analyze these differences further, we extracted quantitative metrics from the time-series data, specifically the RT, PT, FT, and their associated AUCs, AUCR, AUCP, and AUCF.

These signal characteristics were reflected in the extracted parameters summarized in Table 2. The system consistently measured higher AUCP values and shorter RTs in SLE-affected joints compared with the healthy baseline. This trend of shorter RT in diseased joints is consistent with our previously reported results using the FOIS [20]. Additionally, the LOTIS captured a generally shorter and more variable FT in the SLE group compared to healthy controls, reflecting the rapid decay observed in the blunted waveforms.

These representative ranges illustrate the quantitative descriptors accessible using LOTIS; however, formal statistical evaluation of group differences will be addressed in future, adequately powered clinical studies.

The present measurements were conducted on a small cohort and are intended solely to demonstrate the feasibility and physiological plausibility of the LOTIS. The results therefore should not be interpreted as establishing diagnostic performance. Larger clinical studies will be required to determine the sensitivity, specificity, and potential correlations with established clinical disease activity scores.

## 4. Conclusions

In this work we introduced LOTIS, a patient-friendly real-time diffuse optical tomography system designed for quantitative imaging of joint hemodynamics in systemic lupus erythematosus. We detailed the design and fabrication of LOTIS and demonstrated how its improved modular architecture and real-time reconstruction framework address key limitations of the previous-generation system, FOIS, resulting in a more robust and user-friendly imaging platform.

The adoption of a modular hybrid-flex architecture resolves the mechanical stress and component failure points inherent in the original fully flexible design. Signal integrity was substantially enhanced by integrating a 19-bit ADC directly at the optical module, a change that minimizes EMI susceptibility and boosted the mean SNR from 17 dB to 23 dB while also improving the frame rate from 0.75 Hz to 1.25 Hz. The system’s usability was improved with the introduction of multi-finger measurement capabilities, stable 3D-printed attachment rings, and an optimized two-wavelength (660/880 nm) protocol.

A key innovation presented is the development of a real-time, multispectral image reconstruction algorithm based on the SENSOR framework. By integrating a multispectral model and a Discrete Cosine Transform (DCT)-based regularization, we have enabled rapid and accurate reconstructions of clinically relevant chromophore concentrations, such as total hemoglobin, with improved image quality and computational efficiency over traditional methods. This allows for the immediate visualization of joint hemodynamics during measurement.

Our preliminary validation, though based on a small cohort, is highly encouraging. The LOTIS demonstrated the ability to capture qualitatively distinct hemodynamic response patterns of SLE patients and healthy controls. We observed differences in the temporal characteristics of the total hemoglobin response to venous occlusion, with diseased subjects showing a blunted response characterized by shorter rise and fall times. These findings are consistent with our previous studies and reinforce the potential of these dynamic parameters as objective indicators of joint inflammation. However, the present measurements were obtained from a small feasibility cohort and therefore require validation in a larger population. Such studies will allow evaluation of these hemodynamic metrics in relation to established clinical assessments such as joint counts and musculoskeletal ultrasound.

## 5. Patents

Provisional patent application no. 63/931,139 is related to this work.

## Figures and Tables

**Table 1:** Comparison of key specifications between the next-generation Lupus Optical Imaging System (LOTIS) and the first-generation Flexible Optical Imaging System (FOIS).

Feature	LOTIS	FOIS
Light Source	2 LEDs (660 nm, 880 nm)	3 LEDs (530 nm, 655 nm, 940 nm)
Simultaneous Channels	Configurable (Used in a tri-channel configuration)	Single channel only
Frame Rate	1.25 Hz (in tri-channel mode; higher if fewer channels are used)	0.75 Hz
Signal Conditioning	On-module ambient light & digital noise cancellation	No on-board filtering
A/D Conversion	On-module	On a separate control board
Construction	Modular: Rigid sensor PCBs with flexible interconnects	Integrated: Single flexible PCB assembly
Fixation Method	3D-Printed flexible rings	Micropore™ surgical tape
SNR (range, mean)	Range: 18–46 dB; Mean: 23 dB	Range: 7–28 dB; Mean: 17 dB
Inverse Model Computation	<0.2 s (2× speed up via DCT)	Conventional Elementwise
Reconstruction Time	<0.5 s per frame	5 s per frame

**Table 2:** Representative ranges of hemodynamic parameters observed during venous occlusion measurements.

Parameter	Description	Healthy Joints (Representative Range)	SLE Joints (Representative Range)
Rise Time (RT)	Time to 90% peak signal	44–48 s	40–46 s
Plateau Duration (PT)	Duration of peak occlusion	8–13 s	9–16 s
Fall Time (FT)	Time to return to baseline	33–44 s	19–40 s
Rise AUC (AUCR)	Blood volume accumulation	1250–1600	1100–1400
Plateau AUC (AUCP)	Blood volume sustained	470–760	550–880
Fall AUC (AUCF)	Blood volume outflow	2600–3800	1400–3300

## Data Availability

The data presented in this paper can be made available for non-commercial use upon reasonable request, after acceptable completion of the NYU data use agreement. Requests to access the data should be directed to the corresponding author.

## Supplementary Materials

The following supporting information can be downloaded at: https://www.mdpi.com/article/10.3390/bios16040184/s1, Text S1. I2C Address Translation Architecture; Table S1. Address table for I2C address translation for measuring 3 fingers.

## Abbreviations

The following abbreviations are used in this manuscript:

ADC	Analog-to-digital converter
ANC	Ambient Noise Cancellation
AUCF	Area Under the Curve (Fall)
AUCP	Area Under the Curve (Plateau)
AUCR	Area Under the Curve (Rise)
BILAG	British Isles Lupus Assessment Group
CDC	Centers for Disease Control and Prevention
CW	Continuous wave
DCT	Discrete Cosine Transform
DNC	Digital Noise Cancellation
DOT	Diffuse Optical Tomography
EMI	Electromagnetic interference
FIFO	First In First Out Register
FOIS	Flexible Optical Imaging System
FPC	Flexible printed circuit
FT	Fall Time
GUI	Graphical user interface
Hb	Deoxygenated hemoglobin
HbO_2_	Oxygenated hemoglobin
LED	Light-emitting diode
LOTIS	Lupus Optical Tomography Imaging System
MAP	Mean Arterial Pressure
MRI	Magnetic resonance imaging
MSK-US	Musculoskeletal ultrasound
NIR	Near-infrared
PD	Photodiode
PIP	Proximal interphalangeal (joint)
PT	Plateau Time
RA	Rheumatoid arthritis
RT	Rise Time
SD	Source–detector
SENSOR	Sensitivity-Equation-Based Non-Iterative Sparse Optical Reconstruction
SLE	Systemic Lupus Erythematosus
SLEDAI	Systemic lupus erythematosus disease activity index
SoC	System on a chip
SNR	Signal-to-noise ratio
THb	Total hemoglobin
