# Supplementary material for: Patient-Friendly Real-Time Optical Tomographic Imaging System (LOTIS) for Lupus Arthritis
Source: Biosensors (Basel). 2026 Mar 24;16(4):184. doi: 10.3390/bios16040184 (PMC13115400; doi:10.3390/bios16040184)
Supplement: Supplementary file 1 [file biosensors-16-00184-s001.zip › biosensors-4140448-supplementary.pdf]

biosensors

Supplementary Materials

Patient-Friendly Real-Time Optical Tomographic Imaging System (LOTIS) for Lupus Arthritis

Moegammad A. Bardien [1,*,†], Lara Pinar [1,*,†], Alessandro Marone [1], Alberto Nordmann-Gomes [2], Leila Khalili [2], Stephen Suh [2], Stephen H. Kim [1,3,*], Anca D. Askanase [2] and Andreas H. Hielscher [1]

[1] Department of Biomedical Engineering, Tandon School of Engineering, New York University, Brooklyn, NY 11201, USA; am11201@nyu.edu (A.M.); ahh4614@nyu.edu (A.H.H.)
[2] Department of Medicine—Rheumatology, Columbia University, Irving Medical Center, New York, NY 10032, USA; an3363@cumc.columbia.edu (A.N.-G.); lk2482@cumc.columbia.edu (L.K.); sks2231@cumc.columbia.edu (S.S.); ada20@cumc.columbia.edu (A.D.A.)
[3] Department of Medicine—Department of Radiology, Grossman School of Medicine, New York University Langone Health, New York, NY 10016, USA
* Correspondence: mab10058@nyu.edu (M.A.B.); lp2890@nyu.edu (L.P.); hk3363@nyu.edu (S.H.K.)
† These authors contributed equally to this work.

Text S1. I2C Address Translation Architecture

All MAXM86161 modules share a default I2C address of 0x62. To control each module independently, the LOTIS uses I2C address translators (LTC4317) to implement a two-layer addressing scheme that assigns a unique address to every sensor. The first layer of translation occurs on the optical patches themselves. Here, the translator modifies the least significant digit of the base address to give each of the four patches on a finger a unique ID. There are four sets of uniquely assigned patches, which respectively use the address combinations of 0x60 and 0x62; 064 and 0x66; 0x68 and 0x6A; and 0x6C and 0x6E. The second layer of translation occurs on the main control board. This translator modifies the most significant digit of the address to assign a unique ID to each *finger*. For instance, all patch addresses for Finger 1 might be assigned to the 0x6_ range, while Finger 2's patches are moved to the 0x7_ range, and so on. This two-tiered scheme ensures that every module in the system has a unique, predictable address. This hierarchical approach is the key to the system's modularity, as it allows for the rapid setup of single or multi-finger measurements and simplifies maintenance by allowing individual components to be easily swapped. Table S1 summarizes the 2-levels of address translation in the LOTIS.

Table S1. Address table for I2C address translation for measuring 3 fingers.

Finger	Patch No.	MAXM86161 Base Address	Patch level translation	Control board level translation
Finger 1	Patch 1	0x62	0x60	0x60
		0x62	0x62	0x62
	Patch 2	0x62	0x64	0x64
		0x62	0x66	0x66
	Patch 3	0x62	0x68	0x68
		0x62	0x6A	0x6A
	Patch 4	0x62	0x6C	0x6C
		0x62	0x6E	0x6E
Finger 2	Patch 1	0x62	0x60	0x70
		0x62	0x62	0x72
	Patch 2	0x62	0x64	0x74
		0x62	0x66	0x76
	Patch 3	0x62	0x68	0x78
		0x62	0x6A	0x7A
	Patch 4	0x62	0x6C	0x7C
		0x62	0x6E	0x7E
Finger 3	Patch 1	0x62	0x60	0x80
		0x62	0x62	0x82
	Patch 2	0x62	0x64	0x84
		0x62	0x66	0x86
	Patch 3	0x62	0x68	0x88
		0x62	0x6A	0x8A
	Patch 4	0x62	0x6C	0x8C
		0x62	0x6E	0x8E
